# Evidence based recommendations for diagnosis and management of mevalonate kinase defiency (MKD)

**DOI:** 10.1186/1546-0096-12-S1-P73

**Published:** 2014-09-17

**Authors:** Nienke Ter Haar, Jerold Jeyaratnam, Jordi Anton-Lopez, Caroline Galeotti, Karyl Barron, Paul Brogan, Luca Cantarini, Marco Gattorno, Gilles Grateau, Veronique Hentgen, Michael Hofer, Tilmann Kallinich, Isabelle Kone-Paut, Jasmin Kümmerle-Deschner, Helen Lachmann, Huri Ozdogan, Seza Ozen, Yosef Uziel, Carine Wouters, Brian Feldman, Bas Vastert, Nico Wulffraat, Anna Simon, Joost Frenkel

**Affiliations:** 1 Laboratory for Translational Immunology, University Medical Center Utrecht, Utrecht, Netherlands; 2 Department of Pediatrics, University Medical Center Utrecht, Utrecht, Netherlands; 3 Hospital Sant Joan de Déu, Universitat de Barcelona, Barcelona, Spain; 4 Reference Centre for Autoinflammatory Disorders CEREMAI, Bicêtre Hospital, University of Paris SUD, Paris, France; 5 NIH, Bethesda, USA; 6 Department of Rheumatology, UCL Institute of Child Health, London, UK; 7 Policlinico Le Scotte, University of Siena, Siena; 8 G. Gaslini Institute, Genova, Italy; 9 Centre national de référence des amyloses d'origine inflammatoire et de la fièvre , Hôpital Tenon,AP-HP, université Pierre-et-Marie-Curie, Paris, France; 10 Centre Hospitalier de Versailles, Le Chesnay Cedex, France; 11 Department of Pediatrics, University of Lausanne and, University of Geneva, Switzerland; 12 Charite University Medicine, Berlin, Germany; 13 Department of Pediatric Rheumatology, Reference Centre for Autoinflammatory Disorders CEREMAI, Bicêtre Hospital, University of Paris SUD, Paris, France; 14 Klinik für Kinder- und Jugendmedizin, Abteilung für pädiatrische Rheumatologie, Autoinflammation Reference Center Tübingen, Universitätsklinikum Tübingen, Tübingen, Germany; 15 National Amyloidosis Centre, University College London Medical School, London, UK; 16 Cerrahpasa Ic Hastaliklari Klinigi, Istanbul, Turkey; 17 Department of Pediatrics, Hacettepe University Faculty of Medicine, Ankara, Turkey; 18 Department of Pediatrics, Sapir Medical Center, Kfar Saba, Tel-Aviv University, Sackler School of Medicine, Tel-Aviv, Israel; 19 University Hospital Leuven, Leuven, Belgium; 20 Division of Rheumatology, The Hospital for Sick Children, Toronto, Canada; 21 Department of Pediatric Immunology, University Medical Center Utrecht, Utrecht; 22 Department of Medicine, Radboudumc, Nijmegen, Netherlands

## Introduction

Mevalonate kinase deficiency (MKD) is a rare hereditary autoinflammatory syndrome that can lead to significant morbidity. Evidence-based guidelines are lacking and management is mostly based on physician’s experience. Consequently, treatment regimens differ throughout Europe. In 2012, a European initiative called SHARE (*Single Hub and Access point for pediatric Rheumatology in Europe*) was launched to optimize and disseminate diagnostic and management regimens in Europe for children and young adults with rheumatic diseases.

## Objectives

One of the aims of SHARE was to provide evidence based recommendations for diagnosis and treatment of MKD.

## Methods

Evidence based recommendations were developed using the European League Against Rheumatism (EULAR) standard operating procedure. An expert committee was instituted, consisting of pediatric and adult rheumatologists. The expert committee defined search terms for the systematic literature review. Two independent experts scored articles for validity and level of evidence. Recommendations derived from the literature were evaluated by an online survey. Those with less than 80% agreement on the online survey or with relevant comments of the experts were reformulated. Subsequently, all recommendations were discussed at a consensus meeting using the nominal group technique. Recommendations were accepted if more than 80% agreement was reached.

## Results

The literature search yielded 618 articles, of which 28 were considered relevant and therefore scored for validity and level of evidence. Fourteen were scored valid and used in the formulation of the recommendations. Sixteen recommendations were suggested in the online survey and discussed during the consensus meeting. Six general recommendations on management, three recommendations for diagnosis, six for monitoring and seven for treatment were accepted with more than 80% agreement.. Topics covered are the use of the multidisciplinary team, treatment goals, and vaccinations [general recommendations], Gaslini diagnostic score, IgD and urinary mevalonic acid excretion [diagnosis]; the use of AIDAI in clinical studies, monitor frequency, minimal assessments in all MKD and additional assessments in severe MKD patients, infection and macrophage activation syndrome [monitoring], NSAIDs, glucocorticoids, IL-1 blockade, etanercept, switching biologicals, colchicine, statins and hematopoietic stem cell transplantation [treatment].

## Conclusion

The SHARE initiative provides recommendations for diagnosis and treatment for MKD and thereby facilitates improvement and uniformity of care throughout Europe.

## Disclosure of interest

N. Ter Haar Grant / Research Support from: SHARE is funded by the European Commission ( project N° 20111202), J. Jeyaratnam: None declared., J. Anton-Lopez Grant / Research Support from: Abbvie, Novartis, Pfizer, Consultant for: Novartis, Speaker Bureau of: Abbvie, Novartis, Pfizer, Roche, SOBI, C. Galeotti Grant / Research Support from: Novartis, K. Barron: None declared., P. Brogan Grant / Research Support from: Novartis, Roche, Consultant for: Roche, L. Cantarini Grant / Research Support from: Novartis, SOBI, Consultant for: Novartis, SOBI, M. Gattorno Grant / Research Support from: Novartis, Speaker Bureau of: SOBI, G. Grateau Consultant for: Novartis, V. Hentgen Consultant for: Novartis, M. Hofer: None declared., T. Kallinich Grant / Research Support from: Novartis, Speaker Bureau of: Novartis, SOBI, I. Kone-Paut Grant / Research Support from: Chugai, Novartis, SOBI, Consultant for: Abbvie, Chugai, Novartis, Pfizer, SOBI, Speaker Bureau of: Novartis, Pfizer, J. Kümmerle-Deschner Grant / Research Support from: Novartis, Speaker Bureau of: SOBI, H. Lachmann: None declared., H. Ozdogan: None declared., S. Ozen Consultant for: Novartis, Speaker Bureau of: Biovitrium, Y. Uziel Grant / Research Support from: Novartis, Consultant for: Novartis, Speaker Bureau of: Abbvie, Neopharm, Novartis, Roche, C. Wouters: None declared., B. Feldman: None declared., B. Vastert Consultant for: Novartis, N. Wulffraat Grant / Research Support from: Abbvie, GSK, Roche, Consultant for: Genzyme, Novartis, Pfizer, Roche, A. Simon Consultant for: Novartis, Xoma, SOBI, J. Frenkel Consultant for: Novartis, Speaker Bureau of: SOBI.

